# Variable serotonin release assay pattern and specificity of PF4‐specific antibodies in HIT, and clinical relevance

**DOI:** 10.1111/jth.15848

**Published:** 2022-09-02

**Authors:** Jérôme Rollin, Noémie Charuel, Yves Gruel, Sandra Billy, Eve‐Anne Guéry, Marc‐Antoine May, Claire Pouplard, Caroline Vayne

**Affiliations:** ^1^ University of Tours Tours France; ^2^ Department of Haemostasis Regional University Hospital Centre Tours Tours France; ^3^ Department of Cardiovascular Surgery Regional University Hospital Centre Tours Tours France; ^4^ Department of Anesthesiology Regional University Hospital Centre Tours Tours France

**Keywords:** antibody, platelet factor 4, platelets, serotonin release assay, thrombocytopenia, thrombosis

## Abstract

**Background:**

The diagnosis of heparin‐induced thrombocytopenia (HIT) requires functional assays to demonstrate that platelet factor 4 (PF4)‐specific antibodies activate platelets, typically when therapeutic heparin (H) concentrations are tested (“classical” pattern). Some HIT samples also activate platelets without heparin (“atypical” pattern), but with unclear clinical significance.

**Objectives:**

We aimed to assess whether platelet activation pattern and some characteristics of PF4‐specific antibodies were associated with the severity of HIT.

**Patients/Methods:**

Serotonin release assay (SRA) pattern of 81 HIT patients were analyzed and compared with their clinical and biological data, including levels of anti‐PF4/H immunoglobulin G (IgG) and anti‐PF4 IgG in 47 of them.

**Results:**

Higher anti‐PF4/H IgG titers were measured in patients with an “atypical” SRA (optical density 2.52 vs. 1.94 in those with a “classical” pattern, *p* < .001). Patients of both groups had similar platelet count (PC) nadir and time to recovery, but those with an “atypical” SRA more frequently developed thrombotic events (69% vs. 34%, *p* = .037). Significant levels of anti‐PF4 IgG were detected in both groups (38% and 61%, respectively). Whatever the SRA pattern, a lower PC nadir (35 vs. 53 G/L, *p* = .006) and a longer PC recovery time (6 vs. 3 days, *p* = .015) were evidenced in patients with anti‐PF4 antibodies, compared with those with anti‐PF4/H IgG only.

**Conclusions:**

An atypical SRA pattern with elevated anti‐PF4/H IgG titers seems associated with an increased risk of thrombosis in HIT. IgG antibodies to native PF4 may contribute to more severe and persistent thrombocytopenia, and their detection could be useful in clinical practice.

Essentials

- Atypical serotonin release assay (SRA) pattern is frequent in heparin‐induced thrombocytopenia.
- Features of heparin‐induced thrombocytopenia (HIT) antibodies and clinical evolution were analyzed according to the SRA pattern.
- HIT patients with an atypical SRA pattern exhibit an increased risk of thrombosis.
- HIT patients with IgG antibodies to PF4 alone have lower PC nadir and longer PC recovery time.

## 1 INTRODUCTION

Heparin‐induced thrombocytopenia (HIT) is a severe complication of heparin treatment characterized by an atypical immune response, which leads to the development of antibodies (Abs) directed against platelet factor 4 (PF4). Clinical manifestations associated with HIT vary widely, but the most common event is thrombosis,^1^ which results from a multicellular activation involving platelets, monocytes, neutrophils, and endothelial cells.^2^

In most cases, HIT antibodies recognize neoepitopes on PF4 complexed with polyanions such as heparin (PF4/H). These antibodies activate platelets when PF4 and heparin are present in a narrow range of concentrations, which is most often encountered in patients treated with therapeutic or subtherapeutic doses of heparin.^3^ As classically described in HIT functional assays, this results in a “heparin‐dependent” platelet activation pattern, as demonstrated by functional assays performed for HIT confirmation, with a strong platelet activation in the presence of low heparin concentrations, which is absent without or in excess of heparin.

However, it has been known for many years that approximately one‐third to one‐half of HIT samples also strongly activate platelets without requiring heparin in functional assays, whereas complete inhibition of platelet activation is achieved when high concentrations of heparin are present.^4^, ^5^, ^6^, ^7^, ^8^ Beyond a potential role of residual heparin amounts in patients' samples, some studies have also shown that PF4 density on platelets surface and antibody titers could also modulate the capacity of HIT antibodies to activate platelets without heparin *in vitro*.^9^, ^10^ Interestingly, this atypical platelet activation pattern has also recently been associated with the presence of atypical HIT antibodies, able to bind PF4 alone (anti‐PF4 Abs) with very high affinity, and to activate platelet without heparin.^11^, ^12^

Clinically, some authors have also suggested that this atypical platelet activation pattern could be related to more severe HIT syndromes, with deep and persistent thrombocytopenia, a more frequent thrombotic events.^4^, ^6^ However, this concept has never been confirmed by other additional data from a cohort of patients.

In the present study, we assessed the clinical and biological impact of platelet activation pattern observed during functional assays in a cohort of 81 HIT patients. In addition, plasma PF4 concentrations, levels of anti‐PF4/H immunoglobulin G (IgG) and anti‐PF4 IgG antibodies have been measured to further investigate the mechanisms that could explain the atypical platelet activation pattern detected in some patients with HIT.

## 2 PATIENTS AND METHODS

### 2.1 Patients

From May 2000 to October 2021, 109 patients with definite HIT were referred to the University Hospital of Tours (France). All patients had experienced HIT under heparin treatment, with both positive anti‐PF4/H enzyme‐linked immunosorbent assay (ELISA) and serotonin release assay (SRA). Detailed clinical and biological documentation were gathered, including platelet count (PC) evolution during heparin therapy and after its withdrawal. Clinical complications, such as new thrombosis, disseminated intravascular coagulation and bleedings were recorded, as well as sepsis (bacterial presence confirmed) and inflammation (white blood cell count >10 G/L and/or neutrophils count >7.5 G/L and/or C‐reactive protein >5 μg/ml and/or fibrinogen >4 g/L).

The clinical probability of HIT was assessed using the 4Ts score^13^ (calculated retrospectively for HITs before 2006), or the PC evolution pattern after cardiopulmonary bypass and in patients undergoing extracorporeal membrane oxygenation.^14^, ^15^ In these two groups, monophasic and biphasic PC falls were considered as associated with intermediate and high clinical probabilities of HIT, respectively.

Among the 109 patients with confirmed HIT, 68 had a “classical” SRA profile because their plasma samples induced significant platelet activation at low heparin concentrations (0.1–0.5–1 IU/ml), inhibited at high concentration (10 IU/ml), and no significant platelet activation without exogenous heparin (Figure S1). These patients were defined as “classical SRA pattern.”

The 41 other patients exhibited an “atypical” SRA profile, defined by a significant platelet activation (higher than 30%) evidenced even in the absence of exogenous heparin. Of these patients, seven were excluded from the study because no samples were available for further testing, and 21 others because their plasma contained significant residual amounts of heparin (anti‐FXa activity ≥ 0.1 IU/ml), which potentially contributed to the platelet activation observed without exogenous heparin in SRA. Thus, 13 patients were finally included in the “atypical SRA pattern” group.

### 2.2 Blood samples

Blood samples were collected on ethylenediaminetetraacetic acid for PC measurement, and on sodium citrate (0.129 M) for HIT diagnosis assays and standard coagulation tests. Platelet‐poor plasma was isolated by twice successive centrifugations of citrated blood samples (2500*g*, 15 min) and stored at −80°C until assays. This study was approved by the local Ethics Board (DC 2008‐308) and conducted in accordance with the Declaration of Helsinki.

### 2.3 Anti‐PF4/H IgG‐specific ELISA

Considering the large time lapse between inclusions, anti‐PF4/H antibodies were detected at the time of HIT suspicion using several commercial HIT ELISA kits, performed according to the manufacturer's instructions (LIFECODES PF4 Enhanced and LIFECODES PF4 IgG, Immucor GTI Diagnostics, Waukesha, USA; Asserachrom HPIA and Asserachrom HPIA‐IgG, Stago). Therefore, optical density (OD) values obtained could not be rigorously analyzed, and 47 available samples (34 from the classical SRA pattern and 13 from the atypical SRA pattern) were tested again at the time of the study using the same kit (Asserachrom HPIA‐IgG, Stago) to compare anti‐PF4/H IgG titers between the two groups.

### 2.4 Anti‐PF4 IgG‐specific ELISA

IgG antibodies able to bind PF4 alone (anti‐PF4 IgG) were detected in the same 47 patient samples using a homemade ELISA performed as previously described.^16^ Briefly, the plate was coated with 10 μg/ml of human PF4 (HYPHEN Biomed, Neuville‐sur‐Oise, France) at room temperature (RT) overnight. After five washings, bovine serum albumin (5%) was incubated for 2 h at RT. After five another washings, the negative and positive controls and plasma from patients (dilution 1:10) were incubated for 1 h at RT. A goat anti‐human IgG antibody coupled with peroxidase (anti‐kappa murine‐HRP, Jackson ImmunoResearch Europe LTD) was used as a secondary antibody and incubated for 1 h at RT. After washings, the tetramethylbenzidine substrate was incubated for 30 min, the reaction was stopped with H_2_SO_4_ (1 M) and optical densities were read at 450 nm. The cutoff (OD ≥ 0.4) was determined using the mean plus 3 standard deviations of the optical densities measured in 20 healthy donors.

### 2.5 PF4 levels in plasma

Plasma concentrations of human PF4 were measured in the same 47 samples using the Human CXC4/PF4 Immunoassay (R&D Systems), according to the manufacturer's instructions.

### 2.6 Serotonin release assay

All plasma samples were tested at the time of HIT diagnosis using exactly the same SRA,^10^, ^17^ allowing us to compare results obtained over a long period. Only platelets from previously selected healthy subjects with a good responsiveness to HIT antibodies were used. Briefly, whole blood from these platelet donors was collected in acid citrate‐dextrose supplemented with prostaglandin E1 (0.1 mM, Sigma‐Aldrich). Platelet rich plasma was isolated and incubated with ^14^C‐radiolabeled 5‐Hydroxytryptamine binoxalate (PerkinElmer) for 45 min at 37°C. After washings, platelets were incubated with plasma samples and increasing concentrations of unfractionated heparin for 1 h at RT in static conditions. In some experiments, the effects of purified monoclonal antibodies (5B9 or 1E12) previously described^12^, ^18^ were also assessed.

A modified version of the SRA, using exogenous human PF4 (PF4‐SRA), was also performed as described,^10^ with various concentrations of the monoclonal anti‐PF4/H IgG antibody 5B9.^18^ For this purpose, washed platelets were pre‐incubated with human PF4 (10 μg/ml) for 10 min before testing to allow its interaction with the cell surface.

Regardless of the assay performed (conventional SRA or PF4‐SRA), the result was considered positive when a release of ^14^C‐serotonin ≥20% was measured at 0, 0.1, or 0.5 IU/ml of heparin, with complete inhibition at 10 IU/ml (^14^C‐serotonin release <20% or 50% inhibition). Among positive results, an “atypical” SRA pattern was defined by platelet activation ≥30% without exogenous heparin in conventional SRA.

### 2.7 Statistical analysis

The statistical analysis was performed with GraphPad Prism software (version 8.4.0). The quantitative variables were analyzed using a Mann–Whitney test, whereas qualitative variables were analyzed using the exact Fisher test. The Pearson correlation test was carried out to define the cause‐and‐effect relationship of some parameters. When specific data were lacking, the patient was excluded from statistical analysis. A *p* value <.05 was considered statistically significant.

## 3 RESULTS

### 3.1 Biological and clinical features of HIT patients according to their SRA pattern

**FIGURE 1:**
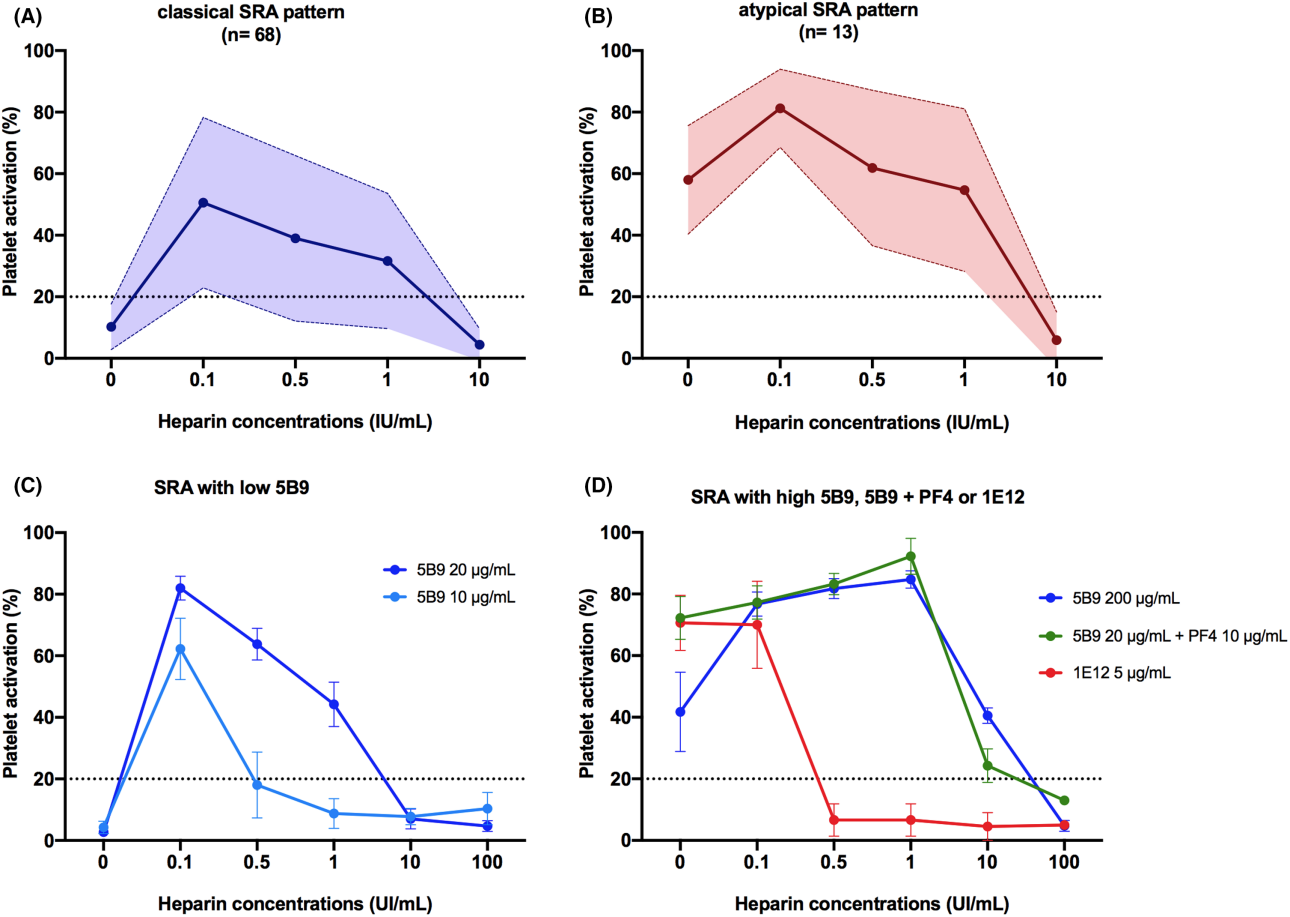
Two types of SRA pattern were observed in our cohort of HIT patients and underlying mechanisms. (A, B) Platelet activation pattern of HIT patients with a classical SRA profile (no platelet activation without heparin, *n* = 34) or an atypical SRA profile (platelet activation without heparin, *n* = 13). Data are mean (%) ± SD. (C) Classical platelet activation pattern observed with the monoclonal anti‐PF4/H IgG antibody 5B9 tested at low concentrations (10 and 20 μg/ml) without and with increasing concentrations of heparin. (D) Three mechanisms contributing to an atypical platelet activation in SRA: high concentrations of anti‐PF4/H IgG, as demonstrated with the monoclonal anti‐PF4/H IgG 5B9 tested at 200 μg/ml; high PF4 concentrations in platelets environment, as shown with 10 μg/ml of exogenous PF4 added to 5B9 20 μg/ml; or the presence of anti‐PF4 IgG antibodies, illustrated here with the monoclonal anti‐PF4 IgG 1E12 at 2.5 μg/ml. In (C, D) data are given as mean ± SEM of *n* = 3 or 4 experiments. HIT, heparin‐induced thrombocytopenia; SRA, serotonin release assay.

Among the 81 enrolled HIT patients, 68 had a classical platelet activation pattern in SRA and 13 others exhibited an atypical SRA profile, associated with a significant platelet activation without heparin (serotonin release ≥30%; Figure 1A,B). In both groups (with classical and atypical SRA pattern), patients were predominantly men, with a median age of 69 years, and treated by heparin in a surgical context in more than 50% of cases (Table 1). There was no difference between the two groups regarding the heparin indication, type of heparin injected, or prior heparin exposure.

**TABLE 1:** Biological and clinical features of the 81 enrolled HIT patients according to their platelet activation pattern in serotonin release assay (SRA)

		Classical SRA profile: no platelet activation without heparin (*n* = 68)	Atypical SRA profile: platelet activation without heparin (*n* = 13)	*p* value
Sex	Males/females	37/31	9/4	NS
Age (y)	Median [min‐max]	69 [21–93]	69 [44–90]	NS
Surgery	*n*	36 (53%)	9 (69%)	NS
Heparin indication	VTE treatment, *n*	9 (13%)	1 (8%)	NS
	VTE prophylaxis, *n*	24 (35%)	7 (54%)	
	Dialysis, *n*	6 (9%)	2 (15%)	
	CPB, *n*	22 (33%)	3 (23%)	
	ECMO, *n*	7 (10%)	0	
Type of heparin	UFH alone, *n*	23 (34%)	3 (25%)	NS
	UFH + LMWH, *n*	32 (47%)	8 (60%)	
	LMWH alone, *n*	13 (19%)	2 (15%)	
Heparin exposure <3 mo	*n*	14 (20%)	1 (8%)	NS
	ND	3	0	
Sepsis	*n*	30 (44%)	8 (62%)	NS
Baseline platelet count (G/L)	Median [min–max]	197 [91–433]	260 [143–372]^a^	NS
Nadir platelet count (G/L)	Median [min–max]	49 [4–134]	38 [6–88]	NS
Time to nadir platelet count (d)	Median [min–max]	10 [3–38]	10 [3–46]	NS
Time to platelet count recovery >150 G/L (d)	Median [min–max]	5 [1–18]^b^	6 [1–21]^c^	NS
Thrombosis	All events, *n*	23 (34%)	9 (69%)	**.037**
	Arterial, *n*	4 (18%)	2 (22%)	NS
	Venous, *n*	14 (61%)	4 (45%)	NS
	Both, *n*	1 (4%)	1 (11%)	NS
	Others events^d^, *n*	4 (17%)	2 (22%)	NS
Other manifestations	DIC, *n*	5 (8.1%)	0	NS
	Bleeding, *n*	16 (23%)	6 (46%)	
Pretest clinical probability of HIT (4Ts score or PC pattern)	Intermediate, *n*	38 (56%)	8 (62%)	NS
	High, *n* (%)	30 (44%)	5 (38%)	
Percentage of platelet activation in SRA	% without heparin, median [min–max]	10 [0–28]	59 [36–92]	**<.001**
	% with low UFH concentrations, median [min–max]	51 [20–100]	87 [57–100]	**<.001**
	% with high UFH concentrations, median [min–max]	3 [0–24]	5 [0–33]	NS

Abbreviations: CPB, cardiopulmonary bypass; DIC, disseminated intravascular coagulation; ECMO, extracorporeal membrane oxygenation; HIT, heparin induced thrombocytopenia; LMWH, low molecular weight heparin; NS, nonsignificant; ND, not determined; PC, platelet count; SRA, serotonin release assay; UFH, unfractionated heparin; VTE, venous thromboembolic event.

^a^ Calculated on 12 patients.

^b^ Calculated on 55 patients.

^c^ Calculated on 11 patients.

^d^ Circuit (dialysis or ECMO circuits) and catheter thrombosis.

Bold values in Tables 1 and 2 indicated p‐values with statistical significance (i.e 〈 0.05).

The baseline PC was similar in both groups, as well as the median PC nadir (49 vs. 38 G/L in classical and atypical SRA groups, respectively, NS) and the time to PC recovery (>150 G/L; median: 5 vs. 6 days, respectively, NS). Importantly, patients with an atypical SRA pattern more frequently developed thrombotic events than those with a classical SRA profile (69% vs. 34%; relative risk = 2.03; *p* = .037). In both groups, all thrombotic events were new and mainly venous. Regarding the other clinical manifestations, disseminated intravascular coagulopathy was present with similar incidences in both groups of patients, and bleedings appeared nearly twice more frequent in those with an atypical SRA pattern, but this difference was not significant.

Interestingly, samples from patients with an atypical SRA profile induced a more potent platelet activation *in vitro*, not only without heparin, but also when low heparin concentrations were tested (median: 87 vs. 51% in classical and atypical SRA groups, respectively, *p* < .001). Of note, all samples from the atypical SRA group induced a serotonin release >50% in the presence of low heparin concentrations, whereas only 50% (34/68) of those from the classical SRA group induced such a high platelet activation *in vitro* (data not shown).

### 3.2 Potential contributors to an atypical SRA pattern

We then investigated the different parameters that may contribute to platelet activation by HIT antibodies without heparin in functional assays. We first defined these parameters using monoclonal PF4‐specific IgG antibodies with a human Fc and then we assessed whether one or more of these mechanisms could explain the platelet activation observed without heparin in our cohort of patients with an atypical SRA profile.

#### 3.2.1 Titers of anti‐PF4/H IgG antibodies

As demonstrated with the monoclonal antibody 5B9, which mimics classical human HIT antibodies,^18^ a high titer of anti‐PF4/H IgG antibodies could explain an atypical SRA profile. Indeed, although 5B9 10 and 20 μg/ml activated platelet only in the presence of low heparin concentrations (Figure 1C), 10 times higher concentrations of this antibody (200 μg/ml) induced platelet activation without heparin in SRA, with a serotonin release greater than 40% (Figure 1D).

**FIGURE 2:**
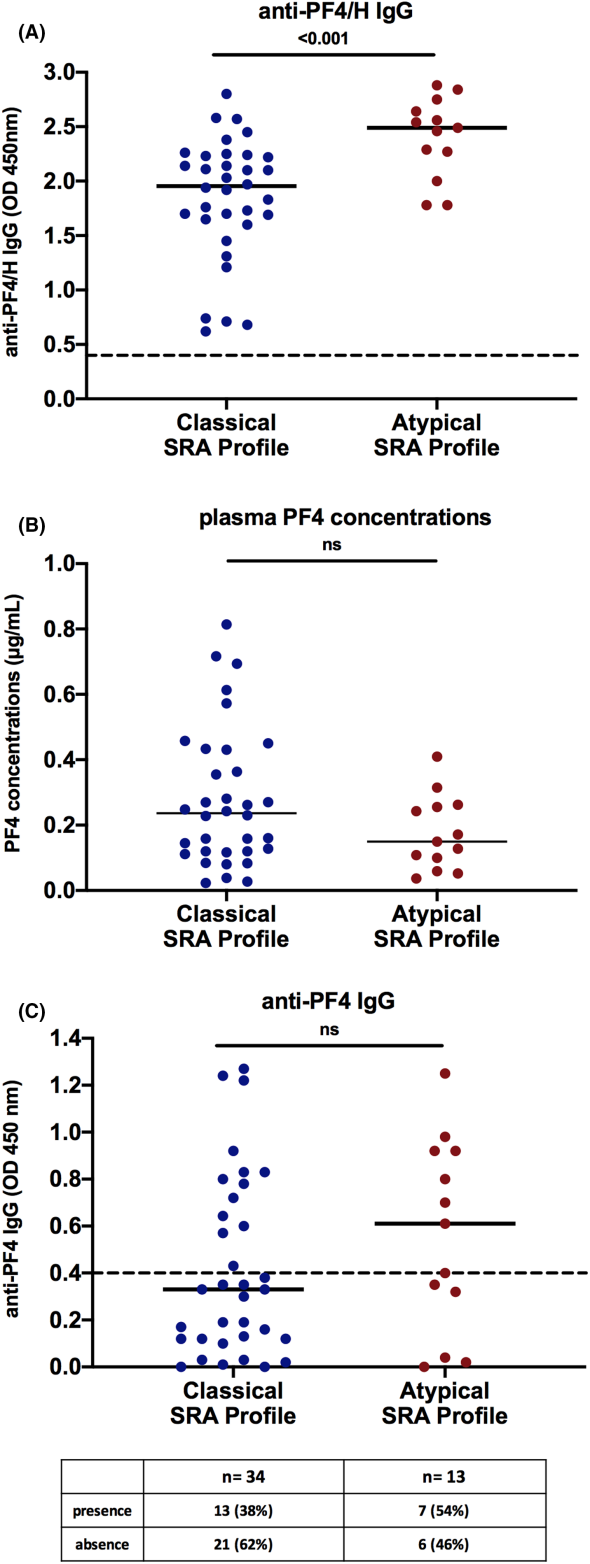
Comparison of anti‐PF4/H IgG titers, plasma PF4 concentrations and anti‐PF4 IgG titers in patients with a classical or an atypical SRA pattern in our cohort of HIT patients. Assessment of anti‐PF4/H IgG titers (A), plasma PF4 concentrations (B), anti‐PF4 IgG titers (C) in HIT patients according to their platelet activation pattern without heparin in SRA (classical profile: no platelet activation, *n* = 34; atypical profile: platelet activation, *n* = 13). Each circle represents a HIT patient, and the solid line indicates the median value of data obtained. Dashed lines in (A and C) represent the cutoff values of each assay. HIT, heparin‐induced thrombocytopenia; SRA, serotonin release assay

In our cohort, different ELISA kits have been used at the time of HIT suspicion, and therefore OD values obtained could not be rigorously compared. For this reason, samples from 34 patients included in the classical SRA group and from 13 with an atypical SRA were tested simultaneously using the same ELISA. Interestingly, higher optical densities were measured with an anti‐PF4/H IgG ELISA in patients with an atypical SRA profile than in those with a classical platelet activation profile (median OD: 2.5 vs. 1.95, respectively, *p* < .001; Figure 2A).

#### 3.2.2 Plasma PF4 concentrations

High PF4 concentrations in the cell environment may also promote the formation of antigenic complexes with chondroitin sulfate moieties present on platelets surface, supporting anti‐PF4/H IgG antibodies binding and platelet activation in the absence of heparin. This concept was supported by the fact that low concentrations of 5B9 (20 μg/ml) activated platelets without heparin in SRA when these cells were preincubated with PF4 (10 μg/ml; Figure 1D).

Nevertheless, PF4 concentrations in our patients were nonsignificantly different in the two groups (median: 0.15 vs. 0.24 μg/ml, in atypical and classical SRA groups, respectively; Figure 2B), and also did not differ from those measured in healthy controls (median: 0.12 μg/ml, min–max: 0.02–0.74 μg/ml; data not shown).

#### 3.2.3 Presence of anti‐PF4 IgG antibodies

We hypothesized that the presence in HIT samples of anti‐PF4 IgG antibodies, could also be associated with an atypical SRA pattern. This was suggested after testing in SRA a low concentration of our monoclonal anti‐PF4 IgG antibody 1E12 (2.5 μg/ml), which strongly activated platelets without heparin,^12^ as confirmed in the present study (Figure 1D).

However, IgG antibodies that bind PF4 alone were detected in our cohort of HIT patients whether they exhibited a classical or atypical SRA profile (Figure 2C). Interestingly, higher titers of these antibodies were measured in those with an atypical SRA profile (median OD: 0.61 vs. 0.33 in the classical SRA group), but this difference was not statistically significant. On the other hand, no correlation was found between anti‐PF4/H and anti‐PF4 IgG titers in both groups of patients (data not shown).

### 3.3 Biological and clinical features of HIT patients according to the detection of anti‐PF4 IgG

In this context, we wondered whether these atypical anti‐PF4 IgG antibodies could affect the biological and clinical features of HIT patients, independently of the SRA pattern.

We therefore defined two groups of patients according to the optical densities measured by ELISA for anti‐PF4 IgG antibodies. Considering a cut‐off value of 0.4, significant levels of anti‐PF4 IgG were evidenced in plasma samples of 21 patients (anti‐PF4 IgG‐positive group), whereas no detectable Abs were found in a second group of 26 patients (anti‐PF4 IgG‐negative group).

**FIGURE 3:**
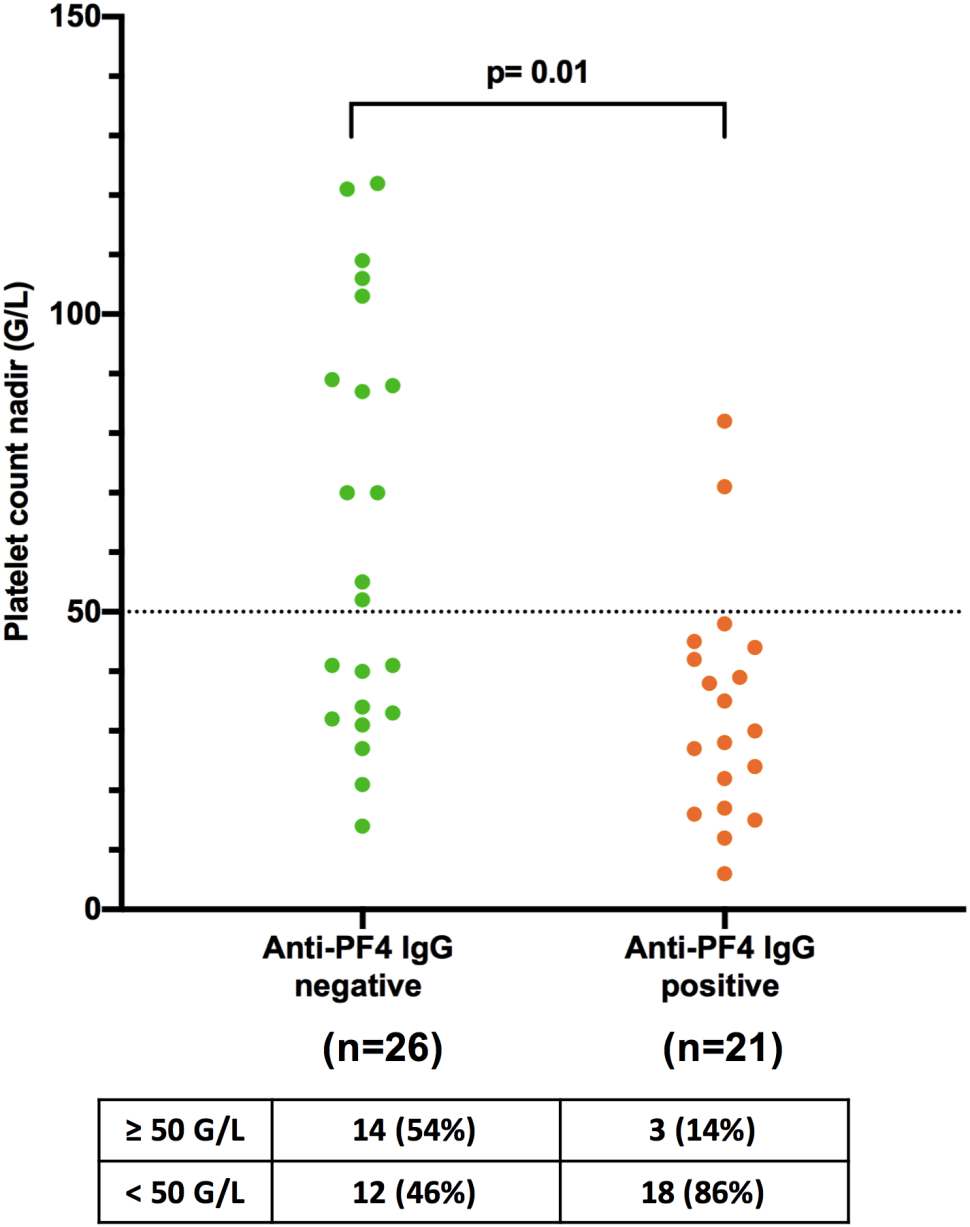
Platelet count nadir in heparin‐induced thrombocytopenia (HIT) patients according to the presence of anti‐PF4 IgG antibodies. Platelet count nadir in patients without (*n* = 26) or with (*n* = 21) detectable anti‐PF4 IgG antibodies. Each circle indicates a HIT patient. The table indicate the proportion of patients (*n*, %) with platelet count nadir below and ≥50 G/L.

After comparing the clinical features of these two groups of patients, we found no significant differences regarding the sex, age, type of heparin injected, indication for heparin treatment, and frequency of surgery, sepsis, or inflammation (Table 2). Platelet activation at low heparin concentrations in SRA appeared stronger in patients with anti‐PF4 IgG (median: 74% vs. 41%, in patients without anti‐PF4 IgG), but the difference observed was not significant. In addition, the incidence of thrombotic events was similar whether patients had developed anti‐PF4 IgG antibodies or not (47.7% vs. 40.9%, respectively). Both groups of patients had similar baseline PCs and time to PC nadir under heparin treatment, but those with detectable anti‐PF4 IgG exhibited a more severe thrombocytopenia, as illustrated by their lower PC nadir (median: 35 G/L vs. 54 G/L in patients with or without anti‐PF4 IgG, respectively, *p* = .006; Table 2) and their higher PC drop (Figure S2). More precisely, 18 of the 21 (86%) patients with detectable anti‐PF4 IgG antibodies had a PC nadir <50 G/L, whereas only 12 of the 26 (46%) patients without anti‐PF4 IgG exhibited such a marked PC decrease (*p* = .01, Figure 3). Of note, PC was lower in patients with anti‐PF4 IgG whether they bled or not (Figure S3). In addition, the time to PC recovery (>150 G/L) after heparin withdrawal in these cases was significantly longer (median: 6 days vs. 3 days in patients without anti‐PF4 IgG, *p* = .015; Table 2), irrespective of their platelet count nadir and the alternative anticoagulant therapy prescribed.

**TABLE 2:** Biological and clinical features of the enrolled HIT patients according to the development of anti‐PF4 IgG antibodies

		Anti‐PF4 IgG negative (*n* = 26)	Anti‐PF4 IgG positive (*n* = 21)	*p* value
Sex	Males/females, *n*	18/8	13/8	NS
Age (y)	Median [min–max]	68 [21–93]	76 [44–90]	NS
Surgery	*n* (%)	17 (66%)	11 (52%)	NS
Heparin indication	VTE treatment, *n*	3 (12%)	2 (10%)	NS
	VTE prophylaxis, *n*	11 (42%)	11 (52%)	
	Dialysis, *n*	0	3 (14%)	
	CPB, *n*	8 (31%)	4 (19%)	
	ECMO, *n*	4 (15%)	1 (5%)	
Type of heparin	UFH alone, *n*	12 (46%)	6 (29%)	NS
	UFH + LMWH, *n*	11 (43%)	11 (52%)	
	LMWH alone, *n*	3 (11%)	4 (19%)	
Heparin exposure <3 mo	*n*	4 (15%)	3 (14%)	NS
	ND	2	0	NS
Sepsis	*n*	13 (52%)	14 (67%)	NS
Baseline platelet count (G/L)	Median [min–max]	194 [114–372]	207 [91–352]^a^	NS
Nadir platelet count (G/L)	Median [min–max]	53 [14–122]	35 [6–82]	**.006**
Baseline‐nadir platelet count drop	Median [min–max]	75 [9–91]	85 [52–97]	**.0053**
Time to nadir platelet count (d)	Median [min–max]	9 [5–22]	10 [3–46]	NS
Time to platelet count recovery > 150 G/L (d)	Median [min–max]	3 [1–21]	6 [2–17]	**.015**
	ND	2	3	
Substitution treatment after heparin discontinuation	Anti–IIa, *n*	8 (31%)	9 (43%)	NS
	Fondaparinux, *n*	5 (19%)	3 (14%)	
	Danaparoid sodium, *n*	12 (46%)	9 (43%)	
	Rivaroxaban, *n*	1 (4%)	0	
Thrombosis	All events, *n*	11 (43%)	9 (43%)	NS
Other manifestations	DIC, *n*	2 (9.1%)	0	NS
	Bleeding, *n*	7 (26%)	11 (52%)	
Pretest clinical probability of HIT (4Ts score or PC pattern)	Intermediate, *n*	17 (65%)	9 (43%)	NS
	High, *n*	9 (35%)	12 (57%)	
Percentage of platelet activation in SRA	% without heparin, median [min–max]	12 [2–92]	14 [0–90]	NS
	% with low UFH concentrations, median [min–max]	41 [20–100]	74 [20–100]	
	% with high UFH concentrations, median [min–max]	4 [0–19]	5 (0.33]	

Abbreviations: CPB, cardiopulmonary bypass; DIC, disseminated intravascular coagulation; ECMO, extracorporeal membrane oxygenation; HIT, heparin induced thrombocytopenia; LMWH, low molecular weight heparin; NS, nonsignificant; ND, not determined; PC, platelet count; PF4, platelet factor 4; SRA, serotonin release assay; UFH, unfractionated heparin; VTE, venous thromboembolic event.

^a^ Calculated on 20 patients.

Bold values in Tables 1 and 2 indicated p‐values with statistical significance (i.e 〈 0.05).

## 4 DISCUSSION

In the present study, we confirmed that patients with HIT frequently exhibit an atypical SRA pattern, with antibodies that significantly activate platelets without heparin. This unusual pattern has been described several years ago,^5^, ^7^, ^19^ especially in “autoimmune HIT”, a rare and heterogeneous entity that includes delayed‐onset HIT, persisting (refractory) HIT, heparin “flush” HIT, and spontaneous HIT.^20^

In half of our patients, there was no residual heparin in their samples, thus supporting that other factors, such as the plasma level and specificity of PF4‐specific IgG antibodies, may explain this atypical SRA pattern.

In our laboratory, we recently obtained different SRA patterns resembling those observed with HIT plasma samples (i.e., either classical or atypical) after testing the effects of two monoclonal PF4‐specific antibodies 5B9 and 1E12, and our results prompted us to evaluate the potential impact of three specific parameters on the platelet activation induced by human PF4‐specific antibodies.

The first parameter is the antibody level, and 5B9, an IgG mimicking classical human HIT antibodies was also able to induce significant platelet activation without heparin when it was tested at very high concentrations. Previously, we showed that patients with high anti‐PF4/H IgG levels were more prompt to exhibit a positive SRA,^21^ and Warkentin et al. also demonstrated a direct relationship between the degree of EIA‐IgG positivity (measured OD) and results of functional assays.^22^ In our study, HIT patients with an atypical SRA pattern had significantly higher titers of anti‐PF4/H IgG, with OD values in EIA always >2.0 except in two cases. Thus, very high titers of anti‐PF4/H IgG antibodies likely contribute to platelet activation without heparin in SRA, similar to that previously suggested in delayed‐onset HIT patients.^4^

The second parameter that could contribute to the ability of some HIT antibodies to activate platelets without heparin *in vitro* is the concentration of PF4 in the cell environment, as suggested by results obtained with 5B9,^10^ and confirmed in this study. However, plasma PF4 concentrations were similar in patients without and with an atypical SRA pattern. Moreover, because these concentrations were below 1 μg/ml and lower than those previously defined for allowing HIT antibodies to induce platelet activation without heparin *in vitro*,^7^, ^9^, ^10^ they unlikely contributed to the atypical pattern of SRA observed in our patients.

The third parameter that could affect the biological activity of HIT antibodies is their specificity. More than 20 years ago, we were the first to report that some patients with classical HIT may develop, apart from antibodies to PF4/H complexes, IgG that bind PF4 alone.^23^ After affinity purification, we also demonstrated that this difference in specificity may influence the ability of HIT samples to activate platelets in SRA.^24^ Lately, Nguyen et al. demonstrated that some patients with an autoimmune HIT had a subset of atypical antibodies that bound PF4 alone (anti‐PF4 IgG) with very high affinity, and strongly activated platelets without heparin.^11^ These features were reproduced with our monoclonal high‐affinity anti‐PF4 IgG, 1E12, but until now the biological and clinical impact of anti‐PF4 IgG had not been assessed in HIT patients. In the present study, IgG antibodies to PF4 alone were associated with a more severe and persistent thrombocytopenia, suggesting they could accelerate platelet clearance.

On the other hand, HIT patients exhibiting a heparin‐independent platelet activation pattern were also more prompt to develop new thrombotic events supporting an association between an atypical SRA pattern and a more severe HIT syndrome.^4^, ^6^ This could be related to higher anti‐PF4/H levels measured in these patients because OD values in ELISA were positively correlated with the extent of platelet activation in functional assays^22^ and the risk of thrombosis in HIT.^25^, ^26^

However, in our cohort, when anti‐PF4/H IgG titers were compared between HIT patients regardless of their SRA profile, similar levels were observed whether or not patients had experienced thrombotic complications (median OD value = 2.1 vs. 1.9, respectively). Therefore, our data support that factors other than anti‐PF4/H IgG titers likely contribute to the risk of thrombosis in HIT patients, including those with an atypical SRA pattern. Interestingly, the extent of platelet activation with therapeutic heparin concentrations in SRA was also higher in this group of patients, reinforcing the hypothesis that they had developed particularly pathogenic antibodies.

Noticeably, our study also has some limitations, which could have affected the data collected and their significance. First, considering the long inclusion time of our patients, the rate of new thrombotic events may have been underestimated because of the use of variable diagnosis strategies and tools. Second, data were analyzed retrospectively, and some HIT samples were not available in sufficient amount to perform in all patients anti‐PF4 IgG EIA and plasma PF4 measurements. Finally, variable sensitivity of donors' platelets to HIT antibodies may have influenced the extent of platelet activation observed in SRA, and potentially the SRA pattern, even though donors were selected for their good reactivity to HIT antibodies.

In conclusion, our study confirms that heparin‐independent platelet activation is relatively frequent in HIT and associated with a higher risk of thrombosis. Moreover, the detection of IgG antibodies that bind to native PF4 could help to identify more severe forms of HIT, but multicenter studies on larger populations of patients are needed to confirm this hypothesis and define the underlying mechanisms involved.

## AUTHOR CONTRIBUTIONS

N.C. performed the research, analyzed data, and wrote the paper; J.R., C.V., and Y.G. designed the research, analyzed data, and wrote the paper; S.B. performed the research and analyzed data; E.A.G. performed the research and analyzed data; M.A.M. contributed to patient recruitment and clinical data collection; and C.P. designed the research and analyzed data. All authors reviewed and approved the final version of the manuscript.

## CONFLICT OF INTEREST

J.R. reports a research grant from Stago. Y.G. reports a research grant and symposium fees from Stago. C.P. reports a research grant from Stago. N.C. is currently an employee of Stago. All other authors of this paper have no conflicts of interest.

## Supporting information


Figure S1‐S3
