# Supplementary material for: Variable serotonin release assay pattern and specificity of PF4‐specific antibodies in HIT, and clinical relevance
Source: J Thromb Haemost. 2022 Sep 2;20(11):2646–55. doi: 10.1111/jth.15848 (PMC9826218; doi:10.1111/jth.15848)

Figure S1: Flowchart of patients' inclusion.

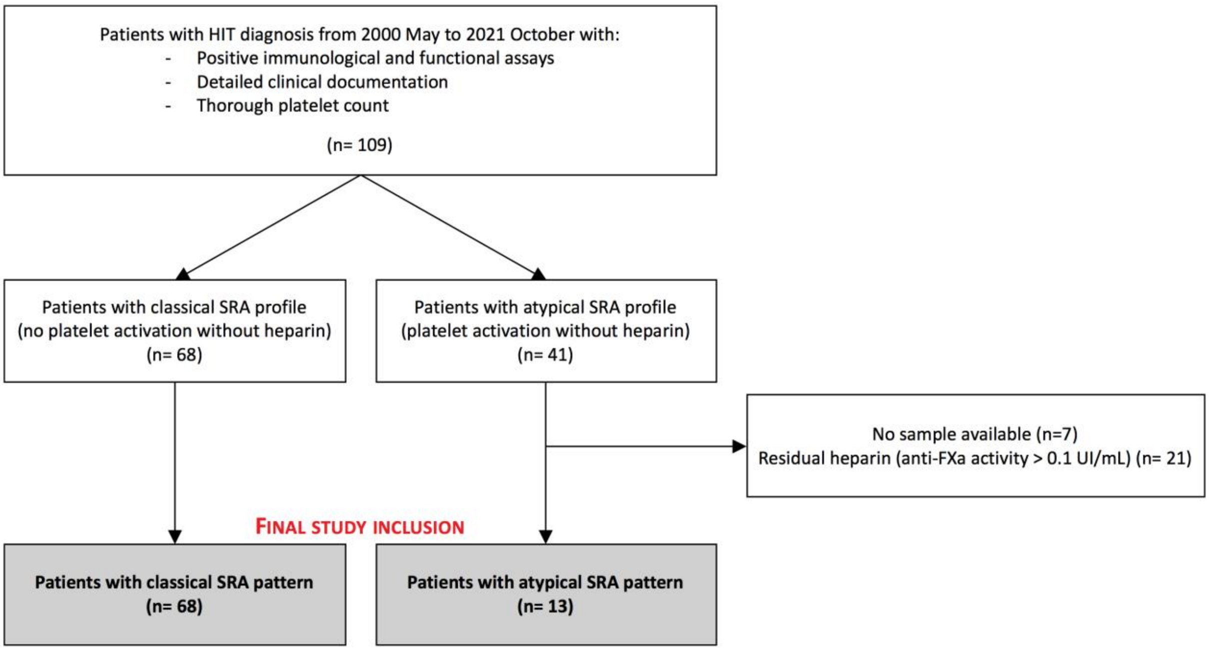

Figure S2: Platelet count drop according to the presence of anti-PF4 IgG in 47 HIT patients.

Platelet count drop in patients without (n= 26) or with (n= 21) detectable anti-PF4 IgG antibodies. Each symbol indicates a HIT patient. Lines represent the median values.

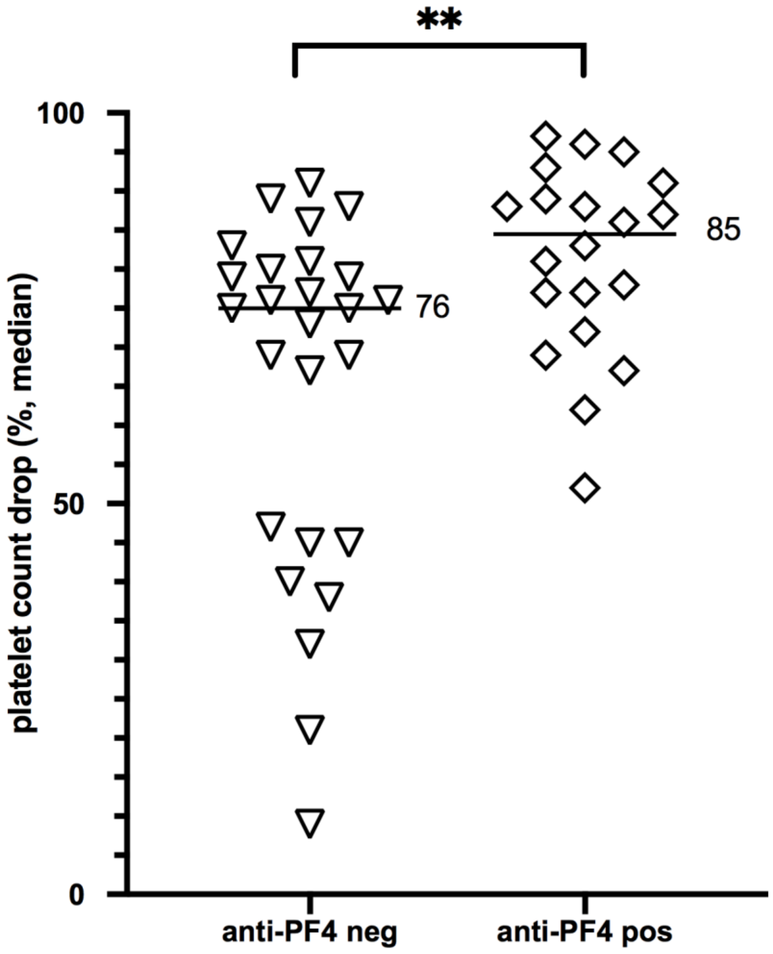

Figure S3: Platelet count nadir according to the presence of anti-PF4 IgG and bleedings in 47 HIT patients.

Platelet count nadir in patients with detectable anti-PF4 IgG antibodies who experienced bleedings (n=11) or not (n=10), and in those without anti-PF4 IgG antibodies, without (n=19) or with (n=7) bleeding complications. Date are presented as box plot (median; min-max).

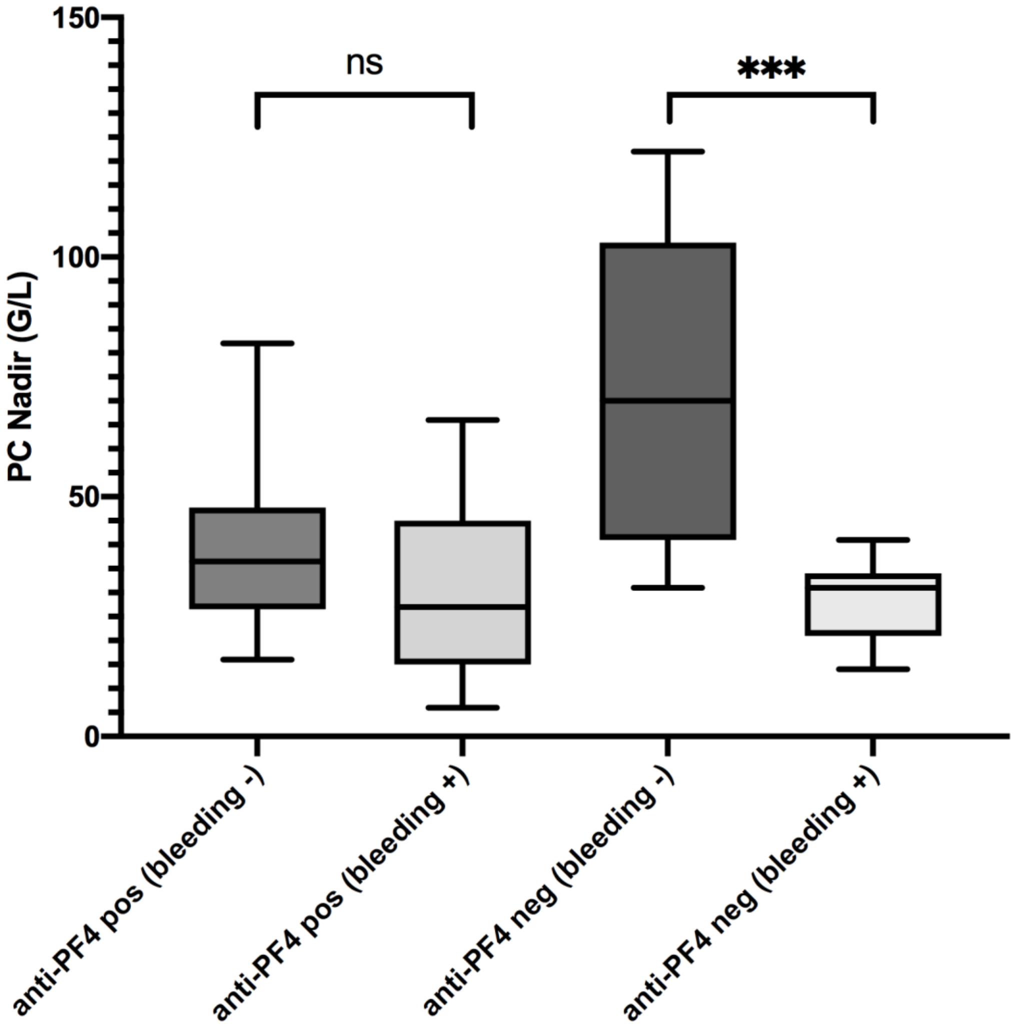

Supplement: Supplementary file 1 — Figure S1‐S3 [file JTH-20-2646-s001.pdf]
